# Identifying Care Home Residents in Electronic Health Records - An OpenSAFELY Short Data Report

**DOI:** 10.12688/wellcomeopenres.16737.1

**Published:** 2021-04-27

**Authors:** Anna Schultze, Chris Bates, Jonathan Cockburn, Brian MacKenna, Emily Nightingale, Helen J Curtis, William J Hulme, Caroline E Morton, Richard Croker, Seb Bacon, Helen I McDonald, Christopher T Rentsch, Krishnan Bhaskaran, Rohini Mathur, Laurie A Tomlinson, Elizabeth J Williamson, Harriet Forbes, John Tazare, Daniel J Grint, Alex J Walker, Peter Inglesby, Nicholas J DeVito, Amir Mehrkar, George Hickman, Simon Davy, Tom Ward, Louis Fisher, David Evans, Kevin Wing, Angel YS Wong, Robert McManus, John Parry, Frank Hester, Sam Harper, Stephen JW Evans, Ian J Douglas, Liam Smeeth, Rosalind M Eggo, Ben Goldacre

**Affiliations:** 11 Electronic Health Records Research Group, Faculty of Epidemiology and Population Health, London School of Hygiene and Tropical Medicine, London, WC1E 7HT, UK; 2The Phoenix Partnership, Leeds, LS18 5PX, UK; 3The DataLab, Nuffield Department of Primary Care Health Sciences, University of Oxford, Oxford, OX2 6GG, UK; 4Centre for Mathematical Modelling of Infectious Diseases, London School of Hygiene & Tropical Medicine, London, Select, WC1E 7HT, UK

**Keywords:** Electronic Health Records, Care Homes, Address Linkage

## Abstract

**Background:** Care home residents have been severely affected by the COVID-19 pandemic. Electronic Health Records (EHR) hold significant potential for studying the healthcare needs of this vulnerable population; however, identifying care home residents in EHR is not straightforward. We describe and compare three different methods for identifying care home residents in the newly created OpenSAFELY-TPP data analytics platform.

**Methods: **Working on behalf of NHS England, we identified individuals aged 65 years or older potentially living in a care home on the 1st of February 2020 using (1) a complex address linkage, in which cleaned GP registered addresses were matched to old age care home addresses using data from the Care and Quality Commission (CQC); (2) coded events in the EHR; (3) household identifiers, age and household size to identify households with more than 3 individuals aged 65 years or older as potential care home residents. Raw addresses were not available to the investigators.

**Results: **Of 4,437,286 individuals aged 65 years or older, 2.27% were identified as potential care home residents using the complex address linkage, 1.96% using coded events, 3.13% using household size and age and 3.74% using either of these methods. 53,210 individuals (32.0% of all potential care home residents) were classified as care home residents using all three methods. Address linkage had the largest overlap with the other methods; 93.3% of individuals identified as care home residents using the address linkage were also identified as such using either coded events or household age and size.

**Conclusion: **We have described the partial overlap between three methods for identifying care home residents in EHR, and provide detailed instructions for how to implement these in OpenSAFELY-TPP to support research into the impact of the COVID-19 pandemic on care home residents.

## Introduction

Care homes in the UK have been severely affected by the COVID-19 pandemic, with many experiencing high mortality rates among residents as well as staff. The Office for National Statistics calculations suggest that between March and the end of June 2020 there were 26,600 excess deaths in care homes in England and Wales, 11,700 (44%) of which were not registered as COVID-19 related
^1^. The vulnerability of care home residents to COVID-19 have raised a number of urgent research questions; however, in the UK there is a lack of datasets identifying care home residents
^2,
3^. Despite this, a large number of studies of COVID-19 in the UK care setting have been conducted to date, using primary data collection through national surveys of certain care home providers
^4^, data from specific care home providers’ Electronic Health Records (EHR) systems
^5^ or data linkages without information on the characteristics of individual residents
^6^.

EHR databases offer a potentially valuable additional resource for studying the impact of COVID-19 on care home residents, both due to the detailed medical history data as well as the presence of a comparator group of older adults resident in their own homes. However, identifying care home residency in these data is complex. Before October 2020 there were no specific requirements for GPs to record care home status, and linkage using GP-registered addresses can be difficult to implement as English addresses are not standardised, and there are no automatic checks of entered addresses against the Care and Quality Commission (CQC) registration details. Compounding this challenge, patients’ residential addresses in their GP record may not always be updated swiftly, or at all, when they move into or out of a care setting, for various reasons. Previous researchers have used a combination of different approaches, including using household size and identifiers
^7,
8^, different types of postcode and address linkages
^7,
9–
11
^ and diagnostic codes
^7,
8^ to identify potential care home residents.

OpenSAFELY-TPP is a new secure analytics platform for electronic patient records built by our group on behalf of NHS England to deliver urgent academic and operational research during the pandemic
^12,
13^. Analyses can currently run across all patients’ full raw pseudonymised primary care records at the 40% of English general practices where TPP EHR software is deployed, with patient-level linkage to various sources of secondary care data; code and analysis is shared openly for inspection and re-use. The OpenSAFELY-TPP framework allows analysis of residential status and ascertains who is living in a care home through a variety of methods.

Working on behalf of NHS England, we described and compared the methods currently available for identifying care home residents in OpenSAFELY-TPP. This report is intended to support all researchers and studies carried out in OpenSAFELY-TPP, and those elsewhere working with the same or similar data, to help inform response to the COVID-19 pandemic.

## Methods

### Data source

OpenSAFELY is an analytics platform for conducting analyses on EHR built inside the data centre where the records are already held. This data centre also imports external datasets from other sources, including A&E attendances and hospital admissions from NHS Digital’s Secondary Use Service, and death registrations from the Office for National Statistics (ONS). Data include pseudonymized data such as coded diagnoses, medications and physiological parameters. No free text data are included. More information on available data sources can be found within the OpenSAFELY documentation
^14^. There are various benefits to avoiding off-site extraction of potentially disclosive pseudonymised patient data: it contributes to enhanced privacy and security; analyses can run in near real-time after clinical events are recorded by clinicians; and all actions are logged. In addition, all code for the OpenSAFELY platform and each individual analysis is shared openly for review and re-use by the wider community, and all data management is done in a standardised framework using OpenSAFELY study definitions
^15^. These are formal specifications, written in the Python programming language, of the datasets to be generated from the underlying raw data. This creates a growing library of standardised and validated variable definitions that can be deployed consistently across multiple projects.

### Information governance

OpenSAFELY NHS England is the data controller; TPP is the data processor and the key researchers on OpenSAFELY are acting on behalf of NHS England. This implementation of OpenSAFELY is hosted within the TPP environment which is accredited to the ISO 27001 information security standard and is NHS IG Toolkit compliant;[1,2] patient data has been pseudonymised for analysis and linkage using industry standard cryptographic hashing techniques; all pseudonymised datasets transmitted for linkage onto OpenSAFELY are encrypted; access to the platform is via a virtual private network (VPN) connection, restricted to a small group of researchers; the researchers hold contracts with NHS England and only access the platform to initiate database queries and statistical models; all database activity is logged; only aggregate statistical outputs leave the platform environment following best practice for anonymisation of results such as statistical disclosure control for low cell counts.[3] The OpenSAFELY research platform adheres to the obligations of the UK General Data Protection Regulation (GDPR) and the Data Protection Act 2018. In March 2020, the Secretary of State for Health and Social Care used powers under the UK Health Service (Control of Patient Information) Regulations 2002 (COPI) to require organisations to process confidential patient information for the purposes of protecting public health, providing healthcare services to the public and monitoring and managing the COVID-19 outbreak and incidents of exposure; this sets aside the requirement for patient consent.[4] Taken together, these provide the legal bases to link patient datasets on the OpenSAFELY platform. GP practices, from which the primary care data are obtained, are required to share relevant health information to support the public health response to the pandemic, and have been informed of the OpenSAFELY analytics platform.

This study was approved by the Health Research Authority (REC reference 20/LO/0651) and by the LSHTM Ethics Board (reference 21863).

### Variable definitions

Three core methods have now been developed within OpenSAFELY-TPP to ascertain whether a pseudonymised patient is a care home resident: address linkage with CQC data; household identifier and decision rules based on household size and age; diagnostic and/or consultation code events directly related to care home residency. These are each described in more detail below.

***Address linkage.*** Individuals’ registered addresses are matched to addresses of old-age care homes (active and historically registered) as held by
CQC. The task is carried out in the secure data centres of TPP and a variable indicating care home status is made available - raw addresses are never available to users of OpenSAFELY-TPP. The exact algorithm for the matching is described on
github. Briefly: CQC care home postcodes are matched on postcode to OpenSAFELY-TPP patients with a valid postcode in their address. This identifies a superset of individuals who live in a postcode which has a care home and will include non-care home residents who share that postcode.

Matches are further refined by the following:

a. *House name matches*. Simple natural language processing is applied to building names within the CQC database to allow matching to the ‘house name’ field in the TPP recorded address. The matching is also applied to other text fields in the address. Individuals within a care home postcode who match a care home house name are considered resident in a care home.b. *House number matches*. Extract street numbers are extracted from CQC and EHR data. If >= 10 addresses are identified on a building name alone, that also have a house number set, then that house number is taken to identify a care home. Individuals within a care home postcode who match a care home house number are considered resident in a care home.c. *Household size matches*. If there are 10+ people aged 65+ on the same address in a CQC postcode for an elderly care home, these individuals are considered resident in a care home. This will miss smaller care homes, but likely results in higher data quality as the risk of error increases the smaller the household size requirement is.

Only individuals with a successful postcode match who also match either criteria 3a, 3b or 3c are considered resident in a care home. Date filters are applied so that only active care homes are matched to active addresses. Finally, CQC metadata on the type of care home (i.e., whether the home provides nursing care or not) is added to addresses.

As highlighted in the code snippet in
Box 1, the current address linkage results in a variable indicating whether a person is a care home resident as of a specific date, with the date supplied by the user of OpenSAFELY -TPP. It is important to note that individuals not resident in care homes may still be resident in other institutional living arrangements, including prisons. When implementing the address linkage, we recommend restricting the variable for care home residency to individuals aged 65 or older, as only old age care homes are included in the TPP address linkage. Younger persons with the care home flag may be live-in carers, family members or individuals from similar addresses that have been incorrectly linked.


Box 1. Identifying care home residents using the TPP address linkage method in the OpenSAFELY study definition frameworkThe address linkage care home status can be accessed through the study definition using the following code:

                    tpp_care_home_type=patients.care_home_status_as_of(
       "index_date",
       categorised_as={
         "care_home_no_nursing": """
          IsPotentialCareHome
          AND LocationDoesNotRequireNursing='Y'
          AND LocationRequiresNursing='N'
         """,
         "care_home_nursing: """
          IsPotentialCareHome
          AND LocationDoesNotRequireNursing='N'
          AND LocationRequiresNursing='Y'
         """,
         "care_home_misc": "IsPotentialCareHome",
         "private_home": "",
      },
      return_expectations={
        "rate": "universal",
        "category": {"ratios": {"PC": 0.05, "PN": 0.05, "PS": 0.05, "U": 0.85,},},
      },
    ),
              



***Household identifiers and size.*** TPP’s records contain a pseudonymised household identifier as well as an estimated household size, the derivation of which will be covered in a separate short variable report. Currently, these are only available for a single date (1st February 2020); however, at this time point they can be used to identify individuals in households of a certain size. Previously implemented decision rules (such as three or four individuals above the age of 65 in the same household)
^7^ can be used to identify potential care home residents.

For this evaluation, we considered individuals as potentially living in a care home if they were aged 65 or older and lived in a household with at least three other people also aged 65 or older. The pseudonymised household identifier can also be used to study specific care homes in a pseudonymised manner; however, it should be noted that care homes can be covered by more than one GP practice and as a result, more than one EHR vendor. This is also a challenge for other GP research datasets. Detailed descriptive statistics on proportions of households covered by TPP will be given in a forthcoming short variable report on the OpenSAFELY household variables where this issue is most salient. An overview of coverage in potential care homes identified using the household method among the over 65s can be seen in
Table 1. The extraction of household ID and estimated TPP coverage within the OpenSAFELY-TPP framework is illustrated in
Box 2. 

**Table 1. T1:** TPP coverage in potential are homes among the over 65s.

Percentage of residents estimated to be registered with a TPP practice	Percentage of households with at least three residents aged 65 or older
>= 75	86.5
>= 80	82.9
>= 85	77.8
>= 90	71.6
>= 95	63.9


Box 2. Identifying care home residents using household identifiers in the OpenSAFELY study definition frameworkThe household identifier can be accessed through the study definition as follows:

                
household_id=patients.household_as_of(
   "index_date",
   returning="pseudo_id",
   return_expectations={
      "int": {"distribution": "normal", "mean": 1000, "stddev": 200},
      "incidence": 1,
   },
),
              
Users should be aware that individuals with invalid or missing household IDs are assigned a household ID of zero at the time of writing, which will need to be handled appropriately before this variable is further use. The percentage of a household which is estimated to be registered with a TPP practice is accessible in as shown below.

                
# mixed household flag
nontpp_household=patients.household_as_of(
    "index_date",
    returning="has_members_in_other_ehr_systems",
    return_expectations={ "incidence": 0.75
    },
),  
# mixed household percentage
tpp_coverage=patients.household_as_of(
    "index_date",
    returning="percentage_of_members_with_data_in_this_backend",
    return_expectations={
       "int": {"distribution": "normal", "mean": 75, "stddev": 10},
       "incidence": 1,
    },
),
              
Once created, users will need to write simple code in whichever programming language they use to identify individuals in households with more than a certain number of individuals over a certain age. 


***Coded events.*** Coded events - such as diagnoses, or consultation location - can be used to indicate whether a certain individual lives or have lived in a care home, or whether a certain consultation occurred in a care home. OpenSAFELY’s codelist tool currently hosts two codelists for identifying care home residents:
a broader list developed by PRIMIS on behalf of PHE to support identification of priority patients for the Covid-19 vaccination programme
^16^; and a
subset of this list containing only two SNOMED codes, which NHS England incentivise general practice and primary care networks to maintain since the 1st of October 2020
^17^ (
Table 2).

**Table 2. T2:** PRIMIS care home codelist.

Code	Term	NHS England Codes?
160734000	Lives in a nursing home	Y
160737007	Lives in an old peoples’ home	N
224224003	Lives in staffed home	N
394923006	Lives in a residential home	Y
248171000000108	Lives in care home	N
1024771000000108	Lives in hospice	N

These codelists can be used to extract information on whether individuals have instances of these codes in their medical records within a given time frame (
Box 3). Different codelists and timeframes can easily be accommodated. For the purposes of these analyses, we considered PRIMIS codelists ever in a patients’ medical record to indicate potential care home residency.


Box 3. Identifying care home residents using coded events in the OpenSAFELY study definition frameworkIndividuals with a diagnostic code indicating care home residency can be identified using the below code in the study definition. The example code illustrates the creation of both an ‘ever’ variable and a ‘within the past year’ variable.

                
#primis codes ever
  primis_carehome_ever=patients.with_these_clinical_events(
     primis_codes,
     on_or_before="index_date",
     returning="binary_flag",
     return_expectations={"incidence": 0.1},
  ),
  
#primis codes within past year
  primis_carehome_pastyear=patients.with_these_clinical_events(
     primis_codes,
     between=["index_date - 1 year", "index_date"],
     returning="binary_flag",
     return_expectations={"incidence": 0.1},
  ),
              



### Analysis

All analyses for this short data report were descriptive in nature and conducted to support our COVID-19 research, where it is frequently important to distinguish care home residents from those living in their own home. We compared the number and percentage of over 65-year-olds identified as care home residents using each of the methodologies described above; these estimates were also compared to national prevalence estimates of care home residency from the 2011 census. Finally, we summarised key characteristics of potential care home residents identified with each different method using descriptive statistics. Data management was performed using Python, with analyses carried out using R version 3.6.2 (2019-12-12). All of the code used for data management and analyses is available at
https://github.com/opensafely/carehomes-short-data-report.

## Results

### Prevalence of potential care home residency in OpenSAFELY-TPP

Among people aged 65 or older registered with a TPP practice on the 1st of February 2020, the three different methods individually identified between 1.96% and 3.13% individuals as potential care home residents (
Table 3). This compares to an estimated prevalence of care home residency among individuals aged 65 or older of 3.2% in the 2011 national census
^18^. As expected, combinations of methods yielded higher estimated prevalence, with a maximum of 3.74% of people identified as potential care home residents by any one of the three methods.

**Table 3. T3:** Prevalence of potential care home residency in OpenSAFELY on the 1 Feb 2020.

Method	N	%
Total (aged >= 65 1 Feb 2020)	4,437,286	100%
Individual methods		
PRIMIS* codelist events (ever)	86,876	1.96%
PRIMIS coded events (past year)	43134	0.97%
Incentivised coded events (ever)	60729	1.37%
Incentivised coded events (past year)	29489	0.66%
Household Size and Age	138,982	3.13%
Address Linkage	100,939	2.27%
Combination of methods		
Address Linkage or Household	152,463	3.44%
Address Linkage or Coded Events	127,928	2.88%
Household or Coded Events	159,041	3.58%
Any of the above	165,845	3.74%

### Overlap between care home residency identification methods

Overlap between the different methodologies can be seen in
Figure 1. Taking individuals identified as potential care home residents using any method (N = 165,845) as the denominator, around a third were consistently identified using all three methods (N = 53,210; 32.1%). 107,742 (65.0%) were identified using at least two different methods.

**Figure 1. f1:**
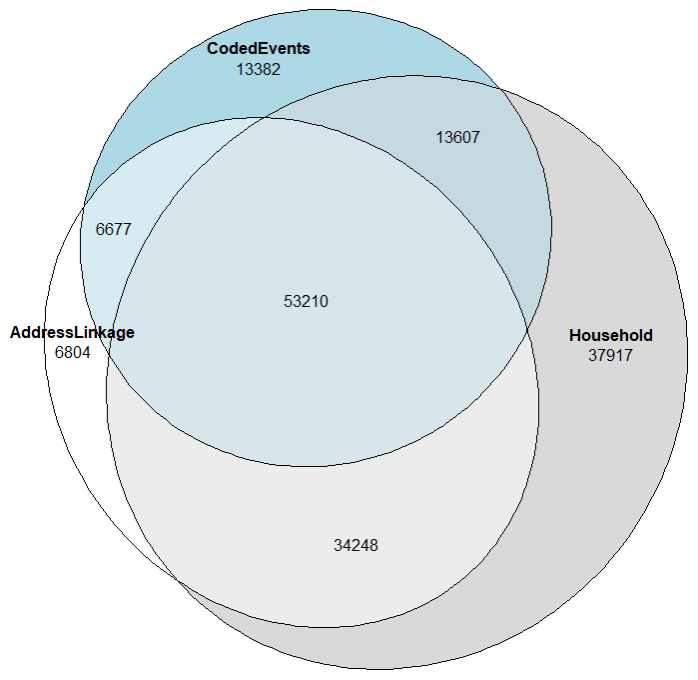
Overlap of care home residency identification using different methodologies.

The address linkage method had the greatest overlap with other methods, 93.3% of individuals identified as potentially resident in a care home using this method were also identified as such by coded events, or through the household method (
Figure 2). However, it also missed a lot of those identified through the “household’ classifier. 52% of potential care home residents identified using the household method and 41% of potential care home residents identified using the address linkage lacked coded events ever in their medical record on the 1st of Feb 2020.

**Figure 2. f2:**
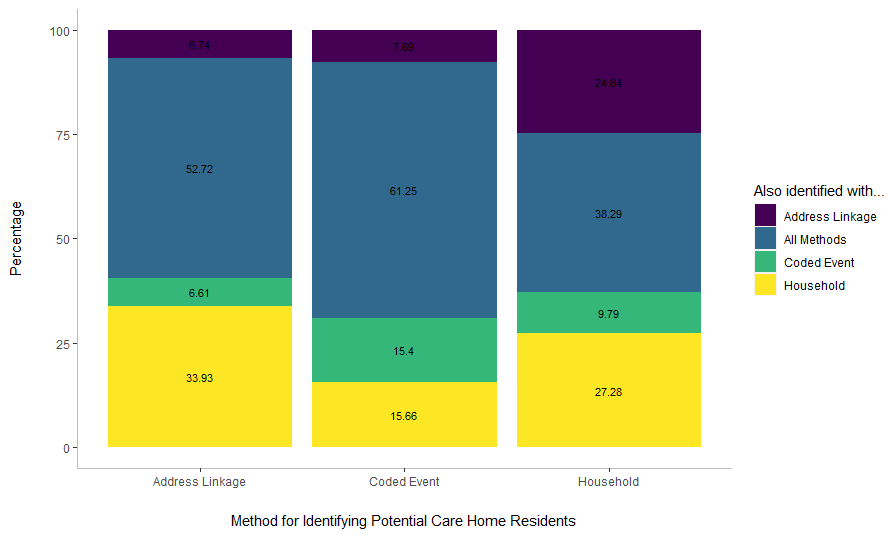
Percentage of potential care home residents identified using each method.

### Characteristics of potential care home residents

To enable a comparison of the characteristics of potential care home residents in OpenSAFELY with data from other sources, we summarised brief demographic and clinical characteristics of individuals identified as a potential care home resident using each method (
Table 4). As expected, the percentage of individuals who were female, over 80 and had a history of dementia was very substantially higher for probable care home residents compared to all over 65s regardless of which method was used to ascertain care home residency status. In terms of age and medical history (dementia and stroke only), individuals identified using the address linkage appeared to be more frail compared to those identified using other methods.

**Table 4. T4:** Key characteristics of care home residents.

	Total over 65s	Address linkage	Household size and age	PRIMIS coded events ever
	%	%	%	%
Male	46.2	30.1	34.3	30
Over 80	27.2	79.1	66.6	75.8
White	94.2	97.9	94.1	97.8
Dementia	4.83	59.3	47.2	58.4
Stroke	6.68	21.7	18.1	21.7

## Discussion

### Summary

Depending on the methodology used, between 1.9% and 3.1% of those aged 65 or older on the 1st of February 2020 were identified as potentially residing in a care home in OpenSAFELY-TPP, which compares to 3.2% in the 2011 national census. There was moderate overlap between the methods, with 65% of potential care home residents identified by at least two different methods.

### Comparisons to previous work

Several different research groups have used and evaluated a variety of methods for identifying care home residents in EHR data
^7,
8,
11^.

Shah and colleagues (2010), using data from 435,568 patients aged 65 or older in the Health Improvement Network (THIN), used either a Read code for care home residency or at least two other care home residence markers (postcode linkage, household size identifier [four or more people aged 65 years or over] and location of consultation) to identify care home residents
^7^. The postcode linkage they applied was the equivalent of the first step of the address linkage we describe here, resulting in a flag indicating whether or not a specific individual resided in a care home postcode. Applying this algorithm resulted in an estimated prevalence of care home residency of 2.7% among patients aged 65 years or older. As in our study, a relatively large percentage of potential care home residents identified using care home markers such as postcode linkage and household rules, did not have diagnostic codes indicating care home residency in their medical record (62%).

Burton and colleagues have since shown that the accuracy of address linkages to identify care home residents can be improved when more complex algorithms are applied
^11^. Using manual address adjudication as a reference standard, they found that more complex linkage algorithms - using Phonics or Markov matching - performed better, with estimated positive predictive value (PPV) of 90% or above, compared to a simpler postcode matching (PPV 77 - 85%). The address linkage method we assess here is more complex than simple postcode linkage but the PPV is currently unknown; there may be potential for further improvement by applying some of these methods. However, while iterative improvements in address matching are important and interesting, we note that all methods reliant on this technique assume that the current GP address is accurate, which is not always the case as patients move into and out of a care home setting.

Lastly, Jain
*et al.* (2017) aimed to identify care home residents in EHRs relying on Read codes for place of residency, consultation location and the Clinical Practice Research Datalink (CPRD) derived ‘family number’ to identify households. A care home resident was defined based on the presence of a diagnostic code, consultation code, or based on residency within a household of three or more individuals >= 65 years of age, if their total count was more than the count of individuals aged <65 years. Using this method identified 4.9% of individuals aged >=65 as potential care home residents, slightly higher than census estimates
^8^. A source of false positives here is likely to be the use of a relatively low household size limit of three, which can often arise from patients taking time to register with a new GP when they move, or not informing their existing GP of an address change, commonly leading to spurious periods of apparent co-habitation of unrelated people in a household. For comparison, the TPP care home flag we have assessed here is limited to those with 10 or more residents to avoid this problem, despite also taking into account linkage of the address to an old age care home. The TPP household identifier will, however, be subject to this limitation, although cleaning of this variable using publicly available data on house sales is undertaken.

### Recommendations for OpenSAFELY-TPP users

As in previous studies on methods to identify care home residency, a key limitation of this descriptive investigation is the lack of a gold standard to compare our methods against. This short report therefore cannot determine the most accurate way of identifying care home residents in EHR. As a consequence, the most suitable method for identifying care home residents will depend on the study question, and whether false positives or false negatives are likely to be of greater concern. For most questions currently investigated in OpenSAFELY-TPP, including COVID-19 risk modelling for care homes, having a high PPV has been regarded as more important than the risk of misclassifying some care home residents as private residents. We have therefore used the address linkage method alone, acknowledging this will only identify a subset of care home residents and that temporary care home stays are unlikely to be captured. For other use cases - for example when estimating denominators for evaluating testing and/or vaccination coverage - different considerations may be more relevant.

Although we cannot recommend a single method for every single study, we can offer the following guidance to help researchers decide which method is most appropriate for their research question:

Where the address linkage identifies someone as residing in a care home we believe this is likely to be accurate, given that the majority (>90%) of these individuals were also considered potential care home residents by the household method and/or coded events method. A high PPV (that is, those identified as care home residents are likely to be true care home residents) has also been found for other complex address linkage methods
^11^. However, users should be aware that this method could miss up to a third of permanent care home residents as compared to the 2011 census, such as those in small care homes with fewer than 10 residents, and likely most temporary residents. This likely results in a significant number of false negatives.The household identifier combined with age and household size results in a greater capture of potential care home residents; however, this is currently only available as of a specific time point (1st of Feb 2020). For addressing the experience of care home residents during the first pandemic wave, this may be sufficient and would allow the potential to capture a slightly larger number of potential care home residents. However, the lower limit for the number of residents in the household (we used three here) can be adjusted upwards to reduce the rate of false positives due to outdated addresses commonly found in patient records. It could also be adapted to take into account the number of under-65s in the household as per some previous studies.Using coded events in isolation underestimated the prevalence of care home residence irrespective of the time frame applied. However, use of diagnostic codes may have improved since the start of the NHS England incentive scheme in October 2020 and prior research using broader codelists, including consultation codes, have estimated a higher prevalence of care home residents. However, to our knowledge there have been no validation studies of using coded events in isolation to identify care home residents. If codes are used in a specific study, users may want to consider developing broader code lists, and should be mindful that the time period applied could impact the accuracy of the classification method.None of the above methods are likely to accurately capture temporary care home stays, which might constitute almost 40% of stays
^19^. The NHS England incentivised codes are required to be added for temporary residents (since October 2020), but it is unclear to what extent this is practical, and how/whether these would be removed once the patient moves back to private accommodation. We have not assessed whether usage of these codes increased after the start of the incentive period, and it is unknown for how long the incentives might be in place. Clinical codes based on location of consultation may be better at capturing patients who were temporarily in a care home but did not have the address added to their record, but it may be possible for these isolated event codes to be inaccurate in some cases. If captured, temporary residents may pose difficulties in interpretation in some research where the study period is significantly longer than a typical temporary stay.

Whichever method a researcher chooses to implement, it is important to acknowledge the potential limitations and to consider the potential impact of the inevitable misclassification of care home residents on the study findings.

### Policy implications and interpretation

Other research teams have previously argued for the development of a minimum dataset for the UK care home population
^2,
20^. During COVID-19 we think this should be a national priority. Such a dataset would allow the identification of both temporary and permanent residents at specific points in time in a way that the methods based on GP registered addresses and diagnostic codes evaluated here will never be able to do. Even a sparse dataset - containing only admission and discharge dates from care homes - would be hugely valuable for studying outbreaks, transmission dynamics, vaccine effectiveness and vaccine coverage. The creation of such a database is complex, but necessary to enable research on the health experience and needs of the UK care home population during the pandemic as well as after
^20^. NHS Digital has recently announced the establishment of a collection of data from adult social care from local authorities who fund a proportion of care home care in England, and the fields in the pilot are likely to assist with ascertaining those who are receiving publicly funded care. 

However, until such a resource has been developed, made available and its quality and character assessed, validation studies of care home status - although potentially complex to design and execute
^21^ - may be warranted. Validation studies of care home residency are particularly complex both due to the lack of a gold-standard and due to the changing nature of care home residency over time. A sample gold-standard list of care home residents from a sample of care homes covered by a single GP EHR provider could in theory be created, allowing estimates of the sensitivity of different care home identification methods at given points in time to be calculated (but not the specificity or predictive values). This might complement existing validation efforts using manual address adjudication as a gold-standard.

### Future work

We are currently investigating the possibility of validating these different methods against a potential gold standard, as well as exploring alternative care home identification methods that could be implemented in OpenSAFELY-TPP - through linkage, refinement of our algorithms, or otherwise. We are keen to receive suggestions on strategies that we may have overlooked.

## Conclusions

We have described three different methods for identifying care home residents in OpenSAFELY-TPP to support ongoing research projects on the impact of the COVID-19 pandemic on this vulnerable population. Although we have not yet conducted a formal validation study, the overlap between our complex address linkage and the alternative identification methods gives us confidence that this method is likely to have a reasonably high PPV. However, like all existing methods based on GP registered addresses it is likely it will result in false negatives and not capture temporary care home stays.

Until a national data infrastructure for care home episodes has been developed, we encourage other research teams to collaborate to share, compare and where possible validate their methodologies for care home identification in EHR data.

## Data availability

### Underlying data

OpenSAFELY:
https://opensafely.org/


The project contains the following underlying data:

- All data were linked, stored and analysed securely within the OpenSAFELY platform
https://opensafely.org/.
**Data include pseudonymized data such as coded diagnoses, medications and physiological parameters. No free text data are included**. All code is shared openly for review and re-use under MIT open license. Detailed pseudonymised patient data is potentially re-identifiable and therefore not shared.- For security and privacy reasons, OpenSAFELY is very different to other approaches for EHR data analysis. The platform does not give researchers unconstrained access to view large volumes of pseudonymised and disclosive patient data, either via download or via a remote desktop. Instead we have produced a series of open source tools that enable researchers to use flexible, pragmatic, but standardised approaches to process raw electronic health records data into “research ready” datasets, and to check that this has been done correctly, without needing to access the patient data directly. Using this data management framework we also generate bespoke dummy datasets. These dummy datasets are used by researchers to develop analysis code in the open, using GitHub. When their data management and data analysis scripts are capable of running to completion, and passing all tests in the OpenSAFELY framework, they are finally sent through to be executed against the real data inside the secure environment, using the OpenSAFELY jobs runner, inside a container using Docker, without the researcher needing access to that raw potential disclosive pseudonymised data themselves. The non-disclosive summary results output tables, logs, and graphs are then manually reviewed, as in other systems, before release.- As part of building that resource for the community, over the next six months we are working with NHS England to cautiously on-board a small number of external pilot users to develop their analyses on OpenSAFELY. This process is described in further detail on our webpage, here:
https://opensafely.org/onboarding-new-users/.

© University of Oxford for the The DataLab 2021.

## References

1 BETA – Data Security Standards - NHS Digital. NHS Digital.
https://digital.nhs.uk/about-nhs-digital/our-work/nhs-digital-data-and-technology-standards/framework/beta---data-security-standards (accessed 30 Apr 2020).2 Data Security and Protection Toolkit - NHS Digital. NHS Digital.
https://digital.nhs.uk/data-and-information/looking-after-information/data-security-and-information-governance/data-security-and-protection-toolkit (accessed 30 Apr 2020).

## Software availability

Source code available from:
https://github.com/opensafely/carehomes-short-data-report
Archived source code at time of publication:
http://doi.org/10.5281/zenodo.4675682
^22^
License: MIT
