# Supplementary material for: Identifying Care Home Residents in Electronic Health Records - An OpenSAFELY Short Data Report
Source: Wellcome Open Res. 2021 Apr 27;6:90. [Version 1] doi: 10.12688/wellcomeopenres.16737.1 (PMC8374378; doi:10.12688/wellcomeopenres.16737.1)
Supplement: Peer review history

# Peer review: Reviewer response for version 1

**DOI:** 10.21956/wellcomeopenres.18458.r45262

**Published:** 2021-08-18

**Authors:** Jennifer Burton

**Affiliations:** 1 Academic Geriatric Medicine, Institute of Cardiovascular and Medical Sciences, University of Glasgow, Glasgow, UK

With thanks for the opportunity to review this important short-report, highlighting a very challenging methodological concern around the accurate identification of the care home population in routine health data sources. Some suggestions are made for the authors to consider:

1. Abstract – It is difficult to contextualise the findings in the absence of a gold standard. The authors acknowledge these limitations clearly in the discussion, but it would be helpful if they could please add a sentence to the conclusions of the abstract reflecting the need for ongoing work to improve/refine the approach. This work is important to demonstrate progress, but has not yet fully resolved the challenge. An acknowledgement of the need for national data infrastructure for care home episodes would also be helpful as, consistent with the authors conclusions, this would be the most effective solution to the problem.
2. Page 5 address linkage – please clarify what 3a, 3b and 3c are – is this just a, b or c?
3. Page 5 – the authors explain rationale for excluding those under 65 years, however it is important to acknowledge that younger people (aged <65 years) live in care home services, both those specifically for their needs (e.g. learning disability, complex physical disability, mental health problems) or in services designated as for older adults but for which there is no alternative accommodation. There is no way to differentiate younger people who are care home residents from staff who live-in. The risk of misclassifying individuals whose address characteristics may match those of the CQC-registered care address is applicable to both younger and older people.
4. Coded events/Table 2 – is ‘lives in hospice’ included in your definition of potential care home residency and, if so, please can you explain the rationale as hospices are distinct clinical settings?
5. Address linkage – was any work done around partial postcode matching, or is only an exact postcode match accepted?
6. Methods – is there any description or link available to the distribution of TPP practices across England and their characteristics, compared to other software vendors?
7. Table 3 – how do PRISMIS codelist events and incentivised coded events overlap? What is the * denoting in line 4?
8. Figure 2 – please change the colour away from purple or change colour of text, numbers aren’t clearly readable even when printed. This figure is also a bit challenging as designed to make complex information more readily understandable, but needs a bit of thinking about to make sense of. Could the additional proportion achieved by each of the specific methods be at the top of the bar for consistency? On quick viewing you could mistakenly think address linkage is the least useful approach and interpreting coded event and household with the current configuration is difficult to grasp visually.
9. Discussion/interpretation: It would be helpful to acknowledge other innovation and developments in this area including work between Health Foundation and NHS England including use of the Unique Property Reference Number (UPRN) https://www.researchgate.net/publication/350071862_A_Novel_Method_for_Identifying_Care_Home_Residents_in_England_A_Validation_Study?channel=doi&linkId=604f72af299bf1736744ef79&showFulltext=true (available from: https://www.health.org.uk/funding-and-partnerships/our-partnerships/improvement-analytics-unit/iau-resources-for-robust-evaluation). This approach is also being tested in Scotland as a means of identifying households: https://www.scadr.ac.uk/sites/default/files/CURLreport2311%20-%20A%20guide%20to%20CHI-UPRN%20Residential%20Linkage.pdf
10. Discussion/interpretation: This paper from the UK Pandemic Ethics Accelerator also helps articulate the challenges around population identification and may be useful contextual information for readers: https://ukpandemicethics.org/wp-content/uploads/2021/06/Making-older-people-visible-in-care-home-data-by-solving-the-denominator-problem.pdf
11. It is worth explaining why the omission of temporary residency is important in relation to the question of interest. Modern use of care homes for step-down, intermediate care and respite, as well as long-term places of residence are particularly poorly served by existing health-based EHRs for capturing residency as it is often dynamic, with individuals moving in and out of homes, particularly after an acute hospital stay. This is a particular challenge when evaluating the initial pandemic period in the UK, where there was enhanced utilisation of care home beds in this way to maximise acute hospital capacity. The authors comment on page 10 that 1 ^st^ February 2020 may be sufficient for assessing first wave care home residency is less certain in view of the mass movement out of hospital which took place in March and into April. It may be better to acknowledge that this is simply an unknown limitation (as per other research in this period specifically relating to those living in care homes: Grimm *et al.*, 2020 ^1^).

Is the work clearly and accurately presented and does it cite the current literature?

Partly

If applicable, is the statistical analysis and its interpretation appropriate?

Not applicable

Are all the source data underlying the results available to ensure full reproducibility?

Yes

Is the study design appropriate and is the work technically sound?

Yes

Are the conclusions drawn adequately supported by the results?

Partly

Are sufficient details of methods and analysis provided to allow replication by others?

Yes

Reviewer Expertise:

Care homes and improving evidence based organisation of care for older people using data-informed approaches

I confirm that I have read this submission and believe that I have an appropriate level of expertise to confirm that it is of an acceptable scientific standard.

## References

[1] : Hospital admissions from care homes in England during the COVID-19 pandemic: a retrospective, cross-sectional analysis using linked administrative data.*Int J Popul Data Sci*.2020;5(4) : 10.23889/ijpds.v5i4.1663166310.23889/ijpds.v5i4.166334286106PMC8267611

# Peer review: Reviewer response for version 1

**DOI:** 10.21956/wellcomeopenres.18458.r43598

**Published:** 2021-05-17

**Authors:** Wasim Baqir

**Affiliations:** 1 Research and Development, Northumbria Healthcare NHS Foundation Trust, Northumberland, UK; 2 National Pharmacy Lead (Pharmacy Integration), NHS England, Northumberland, UK

A good paper which will add much needed clarity of the issue of identifying care home residents. The authors need to decide who is the intended reader. There are a lot of technical details (e.g. box 1) that may not be relevant to most readers and possibly make this too technical. The recommendations and conclusions are valid.

 Introduction

1. May be worth adding in the the number of care home residents across the country or number of beds, for context.
2. Refs 14 and 15 need to be written out in full.

Methods

1. Not clear who the data controller is - is it NHS E or OpenSafely or both?
2. Address Linkage - need a statement on how accurate/ up to date the CQC data set is.
3. Address linkage - criteria 3a 3b 3c - not clear where the '3' comes from.
4. Not sure what box 1 adds; perhaps remove and leave the link to github.
5. Household identifier - again querying the need for box 2 - same applies for box 3.

Results

1. Minor point re formatting in Table 3 - either remove commas or add to all number.

Discussion

1. A short statement regarding younger patients e.g. those in learning disability care homes - these are likely to be missed.
2. Code events refers to NHS Incentive scheme - this is probably the Enhanced Health in Care Homes DES - please cite.
3. This applies to TPP practices - are authors assured that these results are representative?

Two reference sections - merge.

Is the work clearly and accurately presented and does it cite the current literature?

Yes

If applicable, is the statistical analysis and its interpretation appropriate?

Not applicable

Are all the source data underlying the results available to ensure full reproducibility?

Yes

Is the study design appropriate and is the work technically sound?

Yes

Are the conclusions drawn adequately supported by the results?

Yes

Are sufficient details of methods and analysis provided to allow replication by others?

Yes

Reviewer Expertise:

From 20187 to 2020 I was the national pharmacy lead for the NHS England programme, Medicines Optimisation in Care Homes.

I confirm that I have read this submission and believe that I have an appropriate level of expertise to confirm that it is of an acceptable scientific standard.
